# Advances on Hydrogels for Oral Science Research

**DOI:** 10.3390/gels8050302

**Published:** 2022-05-15

**Authors:** Shengjia Ye, Bin Wei, Li Zeng

**Affiliations:** 1 Department of Prosthodontics, Shanghai Ninth People’s Hospital, Shanghai Jiao Tong University School of Medicine, Shanghai 200011, China; hello.ada@outlook.com; 2 College of Stomatology, Shanghai Jiao Tong University, National Center for Stomatology, National Clinical Research Center for Oral Diseases, Shanghai Key Laboratory of Stomatology, Shanghai Engineering Research Center of Advanced Dental Technology and Materials, Shanghai 200011, China; 3 Department of Stomatology Special Consultation Clinic, Shanghai Ninth People’s Hospital, Shanghai Jiao Tong University School of Medicine, Shanghai 200011, China

**Keywords:** hydrogel, oral science, tissue regeneration, drug delivery system

## Abstract

Hydrogels are biocompatible polymer systems, which have become a hotspot in biomedical research. As hydrogels mimic the structure of natural extracellular matrices, they are considered as good scaffold materials in the tissue engineering area for repairing dental pulp and periodontal damages. Combined with different kinds of stem cells and growth factors, various hydrogel complexes have played an optimistic role in endodontic and periodontal tissue engineering studies. Further, hydrogels exhibit biological effects in response to external stimuli, which results in hydrogels having a promising application in local drug delivery. This review summarized the advances of hydrogels in oral science research, in the hopes of providing a reference for future applications.

## 1. Introduction

Hydrogel is a polymer network system formed by cross-linking the reaction of monomers and comprised of water-encapsulating networks [1]. It distinguishes itself from other biological materials by its unique characteristics in structure and performance. The polymer network formed by the hydrogel can bind water, which in turn shows good biocompatibility due to the high moisture content [2,3]. When the hydrogel is combined with biological tissue, its swelling property blurs the boundary between the hydrogel and the tissue, reduces the surface tension, and lessens the surface adhesion of cells and proteins, thus reducing the foreign body reactions [4,5]. Friction and mechanical damage to surrounding tissues can be relatively reduced after hydrogels absorbed water.

**Figure 1:**
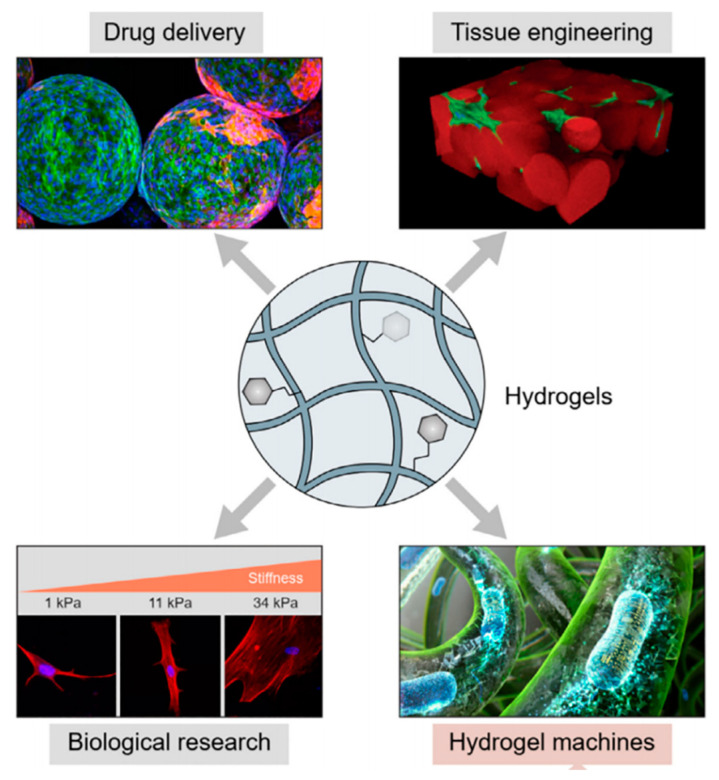
Schematic diagram of hydrogels in tissue engineering (Figure 1 is adapted from reference [10]).

The three-dimensional network structure and viscoelasticity of the hydrogel are similar to the extracellular matrix (ECM), which can mimic the three-dimensional microenvironment of cells, support cells attachment, and induce cells proliferation and differentiation. Because of their favorable properties [3], hydrogels could meet the general requirements of scaffold and drug carriers. Previous studies have shown that hydrogels have been widely applied in biomedical studies of skin, vessels, cartilage, bone, and muscle tissue regeneration [6,7,8,9,10] (Summerized in Figure 1).

Oral health is considered an important part of general health and quality of life [11], and oral disease is still a major public health problem in developed countries and a growing burden for developing countries [12]. Common oral diseases include caries, periodontitis, pulp necrosis, oral mucositis, and so on. Oral science research has developed rapidly in recent years, and hydrogels have become a research hotspot in this field. To exert biological effects accurately and effectively, various hydrogels ranging from natural ones, and synthetic ones to composite hydrogels are being studied [13,14]. This paper reviews the application progress of hydrogels in oral tissue engineering and drug delivery, aiming to provide a reference for the subsequent research and application of biological materials.

## 2. Application of Hydrogels for Dental Pulp Regeneration

Dental pulp, also called endodontium, is located inside the pulp cavity of the tooth. Pulp tissue mainly contains nerves, blood vessels, lymphatic and connective tissues, as well as odontoblasts arranged in the outer periphery of the pulp, whose role is to produce dentin. Dental pulp plays an essential role in the maintenance of blood circulation and homeostasis, sensory transmission, and regeneration of dentin [15]. After conventional root canal treatment due to irreversible pulpitis and pulp necrosis, the pulpless teeth lose their natural biological defense, which may raise the risk of serious caries, apical periodontitis, and ultimately tooth loss [16,17]. Thus, the concept of dental pulp regeneration was put forward to recover the function of teeth and improve the prognosis of a pulpless tooth [14,18].

Pulp regeneration has raised great concerns in the treatment of pulp disease during the past few decades. The American Association of Endodontists (AAE) defines pulp regeneration as, “use biological means to replace damaged dental tissue, root, pulp–dentin complex and other structures to form functional pulp-like tissue”. Studies on pulp regeneration mainly take histoengineering principles and means to induce differentiation of pulp–dentin complex through stem cells–scaffold–growth factor complexes [13], so as to repair damaged pulp tissue and restore physiological functions [14].

The scaffold materials play a variety of roles during this procedure [19], not only limited to providing a three-dimensional structural bracket for cell planting, adhesion, proliferation, and spatial distribution but also regulating cell behavior and intracellular signaling, simulating the recovery of the microenvironment of cell life, and the extra-cellular matrix. The action mechanism of hydrogels meets the mentioned requirements precisely [13,20], they act as carriers of stem/progenitor cells with odontogenic potential [21,22,23,24,25,26,27], carriers of local bioactive molecules [22,28,29,30], and release bioactive factors during degrading [31].

**Figure 2:**
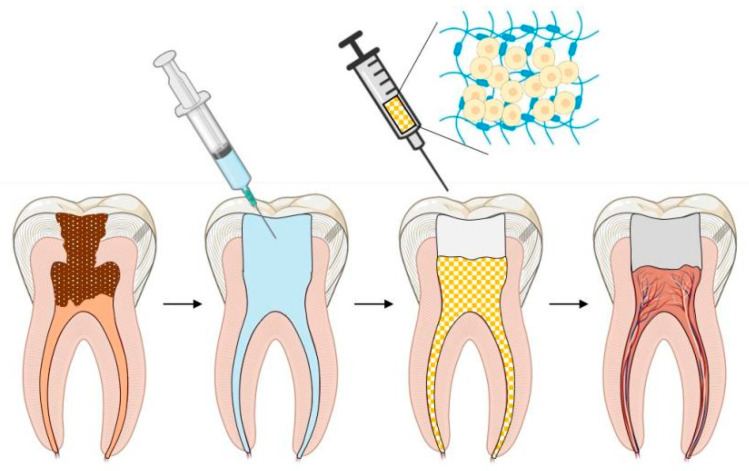
Schematic illustration of ideal pulp regeneration procedure.

Pulp regeneration with hydrogels has become a reality and has been promoted and verified through molecular and developmental biology, as well as biomimetic principles and histological approaches [18,32]. The schematic illustration of the ideal pulp regeneration procedure is shown in Figure 2.

Rosa et al. confirmed that stem cells from exfoliated deciduous teeth (SHED) can generate a functional dental pulp when injected into full-length root canals [26]. SHED survived and began to express specific molecules of odontoblastic differentiation when mixed with commercial peptide hydrogel and recombinant human collagen type I, respectively. Pulp-like tissues were observed with functional odontoblasts throughout the root canals in vivo, presenting similar cellularity and vascularization when compared with control human dental pulps. It appears that the physical properties of the scaffold [33,34], such as viscosity and mechanical capacity, play an important role in dental pulp tissue regeneration. A co-culture of dental pulp stem cells (DPSCs) and human umbilical vein endothelial cells (HUVECs) resulted in the formation of micro-vessels in the bio-printed collagen hydrogel structure within 2 weeks of in vitro culture. Excellent biocompatibility made collagen gel a good choice for the scaffold, while a potential drawback of this hydrogel is shrinkage and rapid degradation in vivo [35].

Chrepa et al. tested the hypothesis that a Food and Drug Administration-approved hyaluronic acid-based injectable gel may be a promising scaffold material for regenerative endodontics. Improvement of stem cells of the apical papilla (SCAP) survival, mineralization, and differentiation into an odontoblastic phenotype was observed in this research [36].

Chitosan, a natural biopolymer derived from chitin, was also found to be able to promote the differentiation and proliferation of dental pulp stromal/stem cells (DPSCs) [37]. Feng et al. [38] utilized small 3D porous chitosan scaffolds fabricated by freeze-drying to support neural differentiation of DPSCs in vitro. Chitosan hydrogel exhibits good conductivity and forms a suitable template. However, some other researchers observed that adding the additional chitosan scaffolds in regenerative procedures did not improve the formation of new mineralized tissues along the root canal walls and the pulp–dentin complex [39,40].

RGD-alginate hydrogels significantly enhance cell adhesion and proliferation [41]. An RGD-bearing alginate framework, that is simply shaped, was used to encapsulate DPSCs and HUVECs equally by Bhoj’s team [28]. Adding dual growth factors to co-culture stem cells within RGD-alginate scaffolds led to the creation of micro-environments that significantly enhanced the proliferation of dental pulp stem cell/human umbilical vein endothelial cell combinations.

The above natural hydrogels are biocompatible, biodegradable, and optimistically bioactive with the ability to release bioactive molecules [13,42]. However, natural ones may carry the risk of disease transmission, immune response, batch variation, and poor mechanical properties [43]. Synthetic hydrogels were developed, characterized by easy standardization, large-scale production, adjustable mechanical properties, and microstructure without the risk of disease transmission. Synthetic hydrogels facilitate regeneration when cooperated with biologically active molecules and cell-binding sequences [44].

Currently, injectable composite hydrogels have become a promising application option in pulp tissue engineering [45,46]. UV light-crosslinked gelatin meth-acryloyl (GelMA) hydrogel has been used to create tissue-engineered pre-vascularized dental pulp-like constructs. Injectable GelMA for DPSCs/HUVECs can promote cell adhesion and proliferation, and meanwhile promote angiogenesis [47]. The survival rates of encapsulating dental pulp cells in GelMA were over 80% [48] and 90% [23] in different studies. Although the optical cross-linking procedure may reduce viability, the manufactured GelMA hydrogel combined with hDPSC/HUVECs posed well in the formation of the vasculature [47]. Studies have also shown that HyStem-C, an injectable composite hydrogel synthesized from polyethylene glycol diacrylate-hyaluronic acid-gelatin, also had good compatibility with DPSCs [49].

However, one of the main limitations of hydrogels is the spatial manipulation restriction, i.e., researchers are unable to fully control the organization and interactions of multiple cells, so the overall morphogenesis of tissues cannot be guaranteed totally. Luckily, combined with superior spatial control of 3D cell printing, this problem can be overcome in the near future [50,51]. The 3D cell printing technology will enable researchers to suspend and place various cells in a hydrogel. For instance, researchers can print odontoblasts along the dentin wall while having fibroblasts in the center of the pulp cavity. While the theoretical application of 3D cell printing in pulp tissue regeneration sounds feasible, there has been a lack of evidence so far. Several studies [50,52] have demonstrated the possibility of success in 3D printing capillaries, but in vivo angiogenesis has not been reported in this area. Although there are few in vivo studies currently, several studies have highlighted the potential application of 3D cell printing in pulp regeneration. For example, in a study by Athirasala and his colleagues, they showed that a novel hydrogel consisting of alginate and dentin (algn-dent) can support mouse odontoblast-like cell lines (OD21) [53].

**Figure 3:**
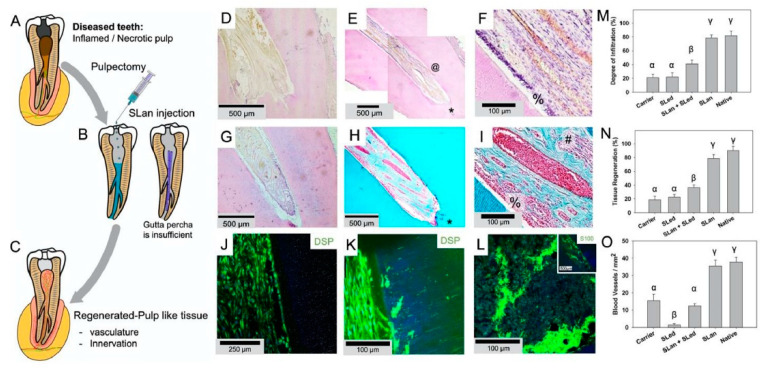
The application of hydrogel in dental root canals in animal model (Figure 3 is adapted from reference [55]). (**A**) Caries and trauma may lead to the inflammation and necrosis of the pulp. (**B**) After pulpectomy, implantation of injectable angiogenic SLan hydrogels help regenerate (**C**) vascularized pulp-like soft tissue in 28 days. In a canine pulpectomy model, disorganized blood clots form for over-instrumentation carrier filled (sucrose-HBSS) control (**D**). H&E staining of tooth roots of SLan filled teeth showed rapid infiltration of cells and tissue (**E**), and within crevices in the canal space (@), along with an odontoblast-like layer in apposition to the dentin wall (**F**-%). Control dentinogenic SLed hydrogels lead to disorganized tissue (**G**). Trichrome staining of SLan implants reveals blood vessels (**H**,**I**) with collagen deposition (blue); and an odontoblast-like layer (**I**-%) which stains with dental sialoprotein (DSP) (**J**) with cytoplasmic protrusions into dentinal tubules (**K**). S100+ Nerve bundles (Trichrome I-#) were regenerated along the length of the canal (**L** and inset). (**M**) Degree of infiltration, (**N**) degree of tissue regeneration, and (**O**) densities of blood vessels were similar for SLan and native teeth but significantly greater than controls.

Currently, in animal models, it appears to be possible to regenerate pulp and dentin, although challenges include the absence of dentin tubular formation, as well as difficulties in dealing with smaller tubes due to angiogenesis [54,55] (Summerized in Figure 3).

The use of growth factors and hydrogel scaffolds accelerates clinical translation and enhances dental tissue engineering, which is expected to be the best biological solution in endodontic medicine [32]. Hydrogel–cell complex-based regenerative endodontics is still in the experimental stage now. AAE (2018) and ESE (2016) have not yet recommended transplanting autologous or allogeneic stem cells in clinical regenerative treatment as the work relates to stem cell isolation, in vitro expansion, good manufacturing practice facilities, stem cell banks, government regulatory issues, clinician skills, training of chairside assistants, and relatively high costs [56].

**Figure 4:**
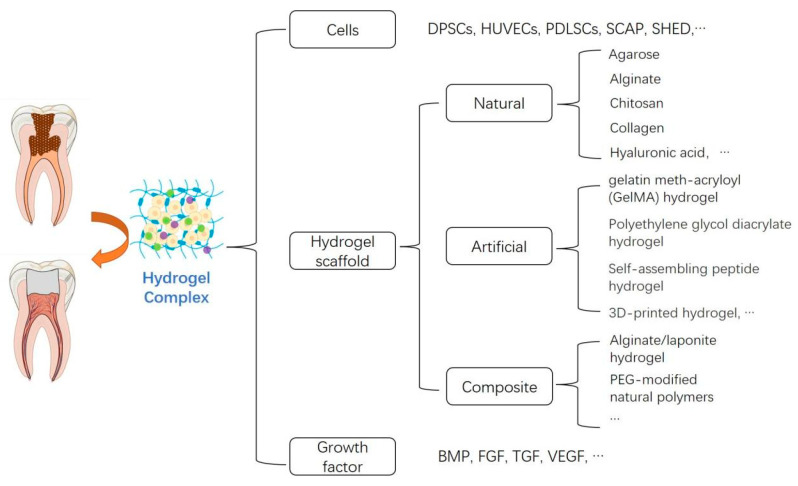
The classification of hydrogel complex in pulp tissue regeneration.

Recent studies suggested that a hydrogel complex [13] may be a strategy to facilitate pulp tissue regeneration (Figure 4). However, in the field of pulp engineering, only a small amount of hydrogels with specific components have been studied in vivo, and there has been no clinical research report so far. In addition, there is still a lack of comparative studies of different hydrogels, further studies are required to enrich current knowledge in pulp tissue regeneration.

## 3. Application of Hydrogels for Periodontal Tissue Regeneration

Periodontal disease is a worldwide health problem that exerts a negative influence on patients. Periodontitis is a chronic inflammatory disease of the periodontal tissue caused by pathogenic microorganisms, with the characteristic of the destruction of teeth supporting structures [57]. Inflammation starts in gums, then penetrates deep, finally resulting in a periodontal pocket of bacteria that erodes the supporting ligaments of the teeth until they are lost [58]. Periodontal diseases lead to certain damage to the nearby tissues, such as loss of attachment, alveolar bone resorption, tooth loosening, etc., which eventually induces tooth loss and endangers oral health and even the whole body [59].

Traditional therapies, including mechanical plaque removal and scaling, are not effective enough in the long term [60]. This invasive method of scaling and root planning (SRP) may result in unpleasant side effects such as sensitivity and tooth topical damage [61]. Classical treatments for periodontitis are time-consuming, technically-sensitive but sub-optimal in the repair of tissue defects [62]. So, alternatives are being looked for in the scientific world. The ideal ultimate therapeutic purpose of periodontal disease is periodontal tissue regeneration to reconstruct both structures and functions.

Some strategies have been conducted to regenerate periodontal tissue, such as the guided tissue/bone regeneration membranes [63]. These applications are promising, while challenges still exist, including low cell transplantation, inaccurate cell localization, immune rejection, difficulty in effectively providing the required growth factors, and inability to control the tissue types that form. Defective areas may be deficient in cells and microvascular formation [1,64]. The main challenge, however, comes from the fact that the periodontal complex is a hybrid tissue unit [1] that consists of highly specialized neural and mechanical receptors, gingiva, alveolar bones, periodontal ligaments (PDL), and cementum. Current regeneration practices focus primarily on the regeneration of individual tissues, unable to simulate and regenerate such complex architectures yet.

**Figure 5:**
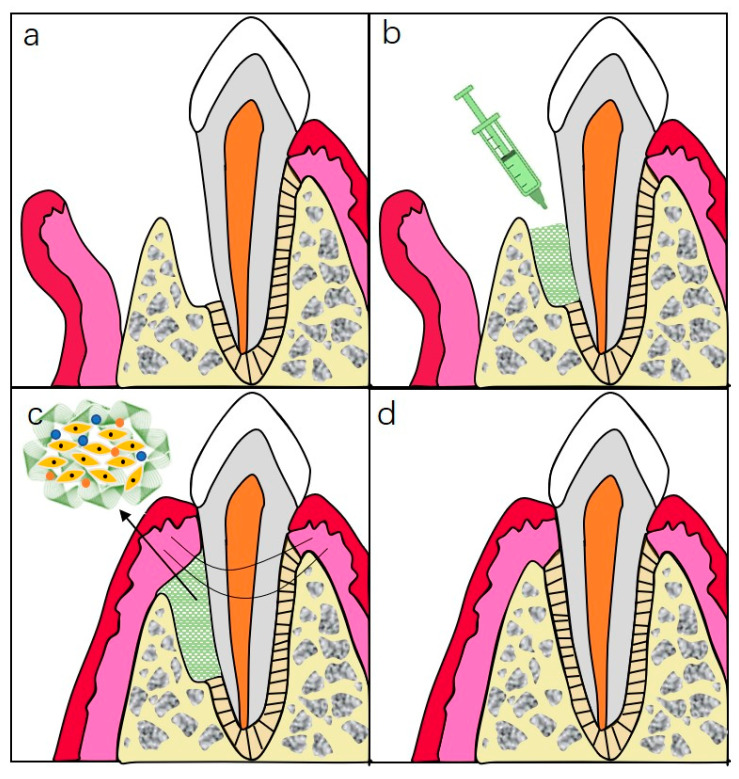
Schematic illustration of injectable hydrogels for periodontal repair. (**a**) Periodontal defect with loss of PDL and alveolar bone. (**b**) Inject hydrogel complexes into the defected site. (**c**) Sewing for closure of the wound. (**d**) Ideal repairment of periodontal tissue.

Recently, hydrogels have been widely applied as a sustained-release system and scaffold materials in periodontal tissue regeneration research [65,66]. While different kinds of hydrogels can be used for dentoalveolar tissue regeneration, their modification or combination is often required for successful strategies [1]. The schematic illustration of the ideal periodontal regeneration procedure is shown in Figure 5.

In the field of alveolar bone regeneration, hydrogels based on hyaluronic acid (HA) have been used with different strategies to augment their mechanical properties. In Miranda’s study, modified hyaluronic acid (HA) and chitosan (CS) were employed to create a hybrid CS-HA hydrogel scaffold [67], which combined the advantages of both ingredients. These porous structures proved suitable for periodontal tissue engineering because the cells migrated more when seeded. Polycaprolactone (mPCL) constructs combined with osteoblasts encapsulated in HA-hydrogel and bone morphogenetic protein-7 (BMP-7) have been proposed in Hamlet’s study, and the constructs were proven to be suitable for mineral deposition in vivo implantation [68].

In addition to cell encapsulation, the combination of GelMA and polyethylene glycol (PEG) has been used for bioprinting regeneration of periodontal tissue [69]. Periodontal ligament stem cells (PDLSCs) encapsulated in this material exhibited higher viability and diffuseness in lower concentrations of PEG, while PEG enhanced the ability to control droplets. In a study on alveolar bone regeneration [70], the further performance of this material was analyzed and its stiffness was observed in the range of 4.5–23.5 kPa, and in vivo analysis results showed bone formation within 6 weeks after implantation. Still, due to the lack of further in-depth descriptions and degradation performance of the structure, structural integrity remains unknown in the long run and needs further studies.

Duarte Campus et al. [71] investigated the effects of the incorporation of collagen in a 3D bio-printed polysaccharide hydrogel on the regulation of cell morphology, osteogenesis potential, and mineralization. The mechanical properties and viscosity increased by combining thermo-responsive agarose hydrogel with collagen type I, which poses a better contour and construct than collagen individually. These composite hydrogels with a high-collagen ratio turned out to be more feasible for mesenchymal stem cells (MSCs) osteogenic differentiation. However, a hydrogel with a compression modulus lower than the natural bone may lead to complications of implant integration, particularly in the load-bearing region [72], which indicates the need to consider adding more mechanically strong materials [73].

PDL, also known as the periodontal ligament, is a highly organized tissue between the cementum and alveolar bone. PDL is capable of taking extremely high forces, which poses a huge challenge for tissue engineering [74]. Constructs combined with hydrogel and stem cells are recommended because of the limited regenerative space. The 3D hydrogel complexes were proposed for a cell-laden array, and the GelMA/PEG composition could be used for periodontal regeneration based on PDLSCs [69]. Yan et al. demonstrated that enzymatically solidified chitosan hydrogels are highly biocompatible and biodegradable. Moreover, chitosan hydrogels without cell loading can improve periodontal regeneration in terms of functional ligament length, indicating the great potential of this hydrogel in clinical applications [75].

A major challenge in periodontal regeneration lies in the complexity [76] of tissue types and variation of repair speed. The introduction of a 3D-printed multiphase scaffold may make the constructs more similar to natural structures with tunable physicochemical and biological characteristics. Lee et al. [77] reported a multiphase matrix produced by bioprinting with different microchannel compartments, which can induce different tissue regeneration as assumed integration. Comprehensive strategies are required in need of regional tissue traits [1], while the network structure and crosslinking process are being dug into to enhance regeneration [78].

Scaffold materials support tissue regeneration to a certain extent, but they may not have the ability to induce tissue regeneration individually. Growth factors, a class of active signaling molecules, can regulate cell growth and other cellular functions by bonding to specific, high-affinity cell membrane receptors. Researchers combined collagen hydrogel scaffold with fibroblast growth factor-2 (FGF2) [79]. This growth factor is able to upregulate cell behaviors and accelerate wound healing to evaluate wound healing in furcation defects in vivo. This application promoted massive cellular and tissue in the growth containing blood vessel-like structure at day 10 and alveolar bone regeneration at 4 weeks. The periodontal attachment was also observed, showing that the FGF2-loaded scaffold was able to guide, reconstruct the function, and self-assemble periodontal organs without abnormal healing.

Chien’s group applied an injectable and thermosensitive chitosan/gelatin/glycerol phosphate hydrogel to provide a 3D environment for transplanted induced pluripotent stem cells (iPSCs) and to enhance stem cell delivery and engraftment [66]. The iPSCs-BMP-6-hydrogel complex promoted osteogenesis, the differentiation of new connective tissue, and the periodontal ligament formation in vivo and reduced the levels of the inflammatory cytokine at the mean time. Hydrogel-encapsulated iPSCs combined with BMP-6 provided a new strategy to enhance periodontal regeneration versatilely.

**Figure 6:**
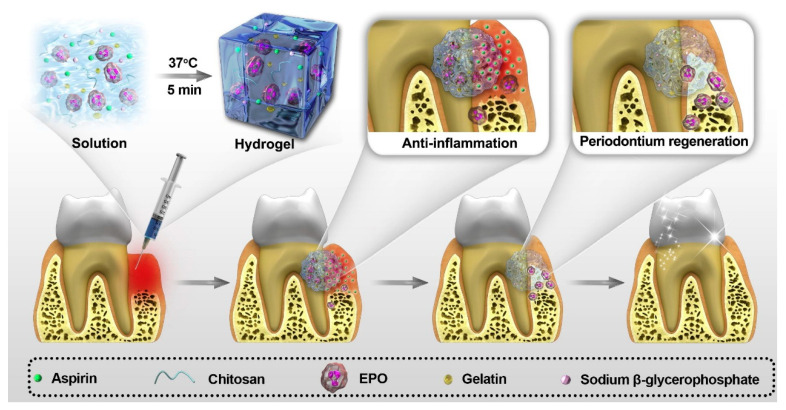
The application of hydrogel complex in periodontal treatment (Figure 6 is adapted from reference [80]).

Xu’s group integrated chitosan, β-sodium glycerophosphate (β-GP), and gelatin to prepare an injectable and thermosensitive hydrogel, which intended to terminate the alveolar bone resorption with simultaneous anti-inflammation and promote periodontium regeneration [80]. The transition occurred at body temperature while seeding in vivo. After being drug-loaded, the hydrogel complex can continuously release aspirin and erythropoietin (EPO) to exert pharmacological effects of anti-inflammation and tissue regeneration, respectively. Both in vitro and in vivo study results demonstrated the potentialities of the hydrogel system in periodontal treatment applications (Smmerized in Figure 6).

A supramolecular hydrogel, SDF-1/BMP-2/NapFFY, was fabricated by combining NapFFY with SDF-1 and BMP-2 recently [81]. It was reported that the two bioactive factors released from the constructs ideally and continuously promote periodontal bone reconstruction both in vitro and in vivo. Specifically, a superior bone reconstruction rate of 56.7% was observed in the treatment of periodontal bone defect model rats after 8 weeks.

In short, periodontal tissue engineering with multiple kinds of hydrogels loaded with various mesenchymal stem cells or bioactive molecules is a promising therapy for an injured periodontal environment. Synthesized hydrogels have great potential for future clinical application, which urges more concerns and investigations in this field. No doubt these novel hydrogels could be able to alter transplantation in the clinic in the near future to repair periodontal defects [32].

## 4. Application of Hydrogels for Drug Delivery in Oral Science

As the common oral cavity diseases locate relatively superficially, the best therapy to control may be regional treatment. Conventional oral drug delivery systems (DDS), such as lozenges and oral spray, work to deliver active drugs topically, while disagreements aroused because of their short residence and instability in saliva [82]. Potential systemic toxicity and low accumulation at target sites are also significant drawbacks of the traditional ones [83,84]. In recent years, new DDSs have attracted the increasing attention of researchers [83,85] because of their ability to provide higher drug absorption and other routes of administration, efficient drug targeting, and lower systemic toxicity.

Different kinds of DDSs are being developed [86] including hydrogel, liposomes [87], electrospun nanofibers, mucoadhesive films, and micelles. A primary defect of the topical therapeutic administration is insufficient residence in the oral cavity. Take liposomal delivery systems, for instance, limitations of instability, drug leakage, and difficulties in large-scale manufacture cannot be ignored [88], although the liposomal antimicrobial agents targeting biofilms have proven effective.

As is depicted in the above text, hydrogels can absorb a large amount of liquid and swell due to their fantastic hydrophilicity, with good viscoelasticity and longer residence time. They are introduced as a novel DDSs to encapsulate various therapeutic agents/compounds and release them in a controlled manner [89]. A recent review discussed the environment-sensitive hydrogels as the “smart” ones, which are able to respond to various multiple stimuli, such as temperature, pH, light, enzymes, pressure, and so on, therefore, it is a promising approach to be used in clinic [90]. Despite releasing effective compounds with a controlled profile by hydrogel complex, some intelligent systems have been fabricated using physical and chemical stimuli as a sensor [91,92]. Temperature-sensitive hydrogels transform from the sol to gel phase at a body temperature of 37 °C [80,93,94,95] and facilitate drug release. Photosensitive hydrogels are supposed to be activated by a certain wavelength of light, generating ROS to kill microorganisms as well as phase transformation [96,97]. Several pH-sensitive drug delivery hydrogels with the ability to swell or shrink in response to pH changes have been reported, where the polymers could either accept or release protons in response to changes in pH in the microenvironment [98,99].

In pathological conditions, specific changes would occur in the local microenvironment of the tissue, such as local pH reduction under various conditions. As a drug delivery carrier, certain hydrogels complexes are fabricated to respond to local pathological stimuli and achieve delivery at a very point, affecting the biological distribution and toxicity of drugs.

Several researchers constructed an agarose hydrogel system for biomimetic mineralization of dentin [100] and enamel [101]. The designed systems displayed a good condition of mineralization in vitro, analyzed with scanning electron microscopy, X-ray diffraction, Fourier transform infrared spectroscopy, and the nanoindentation hardness test. Muşat’s team first reported the simultaneous use of chitosan (CS) and agarose (A) in a biopolymer-based hydrogel for the biomimetic remineralization of an acid-etched native enamel surface [102]. They observed analogous Ca/P compound covered on natural tooth enamel, and found the microhardness recovery of the enamel-like layer under CS-A hydrogels by a 7-day remineralization process in artificial saliva. Ren’s team designed a more clinically powerful anti-caries treatment by combining amelogenin-derived peptide QP5 with antibacterial chitosan in a hydrogel (CS-QP5 hydrogel), and reported an inhibition of cariogenic bacteria and the promotion of remineralization of initial caries lesions [103]. Therefore, these methods provide the experimental basis for remineralization and novel strategies to treat dentin hypersensitivity and dental caries.

Antimicrobial activity improves when hydrogels are loaded with antibiotics [104]. Aksel et al. found that the antibiotic-loaded chitosan-fibrin hydrogel enhanced the antibacterial property against E. faecalis biofilm [105]. Metronidazole and ciprofloxacin-loaded chitosan were found more suitable due to their perfect antibacterial property while maintaining cellular function. Yan et al. applied GelMA hydrogel as a carrier of metronidazole (MTR) and chlorhexidine (CHX) [106], and obvious antimicrobial effects against E. faecalis, S. mutans, and P. intermedia were noticed. A similar application with GelMA and CHX was taken by Ribeiro et al. [107], they formulated injectable chlorhexidine (CHX)-loaded nanotube-modified GelMA hydrogel which provided the sustained release of CHX for dental infection ablation against E. faecalis. Ren et al. [103] designed CS-QP5 hydrogel which has a good antibacterial potency toward Streptococcus mutans by reducing adhesion and biofilm formation. Drug-loaded hydrogels might be a promising material for root canal disinfection and carious treatment to inhibit the dental interest of bacteria.

It is certain that periodontitis initiates from uncontrolled plaque which includes various microorganisms [108]. Bacterial infections are the main reason for the destruction of periodontal tissue. Local medication raised more attention instead of conventional systemic antibiotic therapy [86]. Periodontal sustained-release medications can prolong the duration of drug action and reduce the number of administrations [109,110,111]. An injectable and photo-cross-linkable gelatin methacryloyl (GelMA) hydrogel was engineered with ciprofloxacin (CIP)-eluting short nanofibers for oral infection ablation by Ribeiro et al. [110]. The hydrogels promoted localized, sustained, and effective cell-friendly antibiotic doses, meaning a good efficacy in inhibiting Enterococcus faecalis inflammation. Chang et al. designed a naringin-carrying CHC-β-GP-glycerol colloidal hydrogel [111], which can be used to inhibit experimental periodontitis with favorable handling and inflammation-responsive characteristics. A chitosan membrane containing polyphosphoester and minocycline hydrochloride (PPEM) was prepared in Li’s research [112]. During the progression of the periodontitis, overexpressed ALP will promote the degradation of PPE and the release of antibiotics in the meantime. Liang’s team came up with an optimal formulation of carbomer hydrogel, toluidine blue O (TBO) and NaOH, which improved the therapeutic effect of the original photodynamic therapy against Staphylococcus aureus and Escherichia coli [113]. Therefore, photodynamic therapy with the novel optimized TBO hydrogel formulations can be a promising strategy to treat periodontitis.

Hydrogel administration is conducted by injecting into the infected periodontal pocket [114,115,116], maintaining a controlled and constant concentration of the target drug, which cannot be removed by salivary flush. Side effects will be lessened with interesting potential for endogenous repair of alveolar bone [117].

Oral mucosal diseases such as lichen planus, aphthous stomatitis, oral mucositis, and wounds mostly require effective topical therapies. The primary problem in topical administration of therapeutic agents lies in the low residence time on the smooth and moist surface of oral soft tissue [86].

Hydrogels can be applied in mucosal injury as well for their elastic, adhesive, and degradable characteristics. Andreopoulos et al. [118] reported a method to prepare light-tunable PEG-NC gel scaffolds and the delivery of bFGF from the hydrogels could be controlled by altering the gel properties. They proposed that hydrogels can be applied as a wound healing membrane to treat chronic wounds. Carbomer hydrogels were also proven effective to promote greater residence time on the mucosa when the Carbopol^®^ 980 was combined with lipid nanoparticles (NLC) for buccal administration [119]. Zhang et al. created a photo-triggered hydrogel adhesive [120], which operated on a fast S-nitrosylation coupling reaction and connected to host tissues. This novel hyaluronic acid gel was able to protect mucosal wounds for more than 24 h. The results from animal oral mucosa repair models demonstrated that this hydrogel adhesive created a favorable microenvironment for tissue repair and shortened tissue healing time, illustrating a promising therapy to advance the treatment of oral mucosal defects.

The proposal of a thermally sensitive mucoadhesive hydrogel aimed to facilitate the treatment of oral mucositis, which contained Trimethyl chitosan (TMC) and methylpyrrolidinone chitosan (MPC) [9,121]. Mixed with glycerophosphate (GP) according to different ratios, the best properties were shown. In addition, anti-inflammatory drugs such as benzydamine hydrochloride could be loaded on the complex, which showed good antimicrobial properties.

Antioxidants were also mixed with hydrogels to play roles in the oral cavity, and an isoguanosine–tannic acid (isoG-TA) supramolecular hydrogel was fabricated with leukoplakia (OLK) by Ding et al. [122]. Results showed that the proliferation of dysplastic oral keratinocytes (DOKs) was inhibited due to the antioxidant property of the complex. Azadikhah and his colleagues developed a new antioxidant-photosensitizing hydrogel based on chitosan to control photodynamic therapy (PDT) activity in cancer treatment [123], which help to minimize the damage risk for normal cells. Hesperetin-loaded carbopol hydrogel can also be an effective therapy with a controlled release profile and could be used to treat topical oxidative conditions [124].

**Figure 7:**
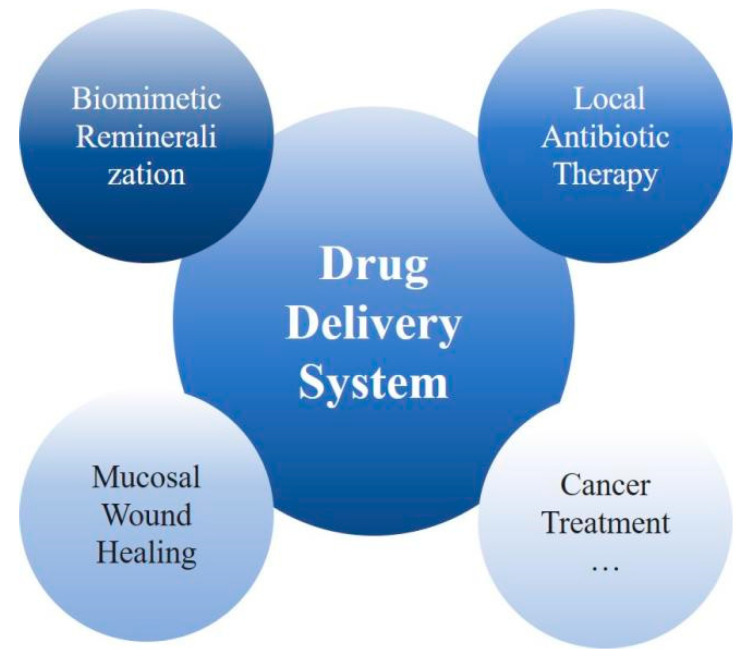
Drug delivery system based on hydrogel application for oral diseases.

In conclusion, there are various formulations based on hydrogels in DDSs. Figure 7 illustrates the scope of the system in oral diseases. The advantages of such treatment are manifold, because they directly target the affected area, maintain relatively constant drug concentration levels, minimize systemic side effects as well as improve patient compliance.

## 5. Conclusions

This article reviews the potential of hydrogel to treat pathogenic oral cavity conditions. The applications of hydrogels for oral science research are wide, ranging from tissue reconstruction to oral disease therapy. The advantages of the application of hydrogel complexes include physical property [125], the straightforward chemistry procedure [126], ease of dental-derived MSC load, partially being condition-responsive, injectability, biodegradability, and the introduction of a three-dimensional delivery scaffold for tissue engineering. Although the results of most studies were promising, a larger number of clinical studies for determining the efficiency of prepared systems is required.
